# Decreased level of recent thymic emigrants in CD4+ and CD8+T cells from CML patients

**DOI:** 10.1186/1479-5876-8-47

**Published:** 2010-05-14

**Authors:** Yangqiu Li, Suxia Geng, Qingsong Yin, Shaohua Chen, Lijian Yang, Xiuli Wu, Bo Li, Xin Du, Christian A Schmidt, Grzegorz K Przybylski

**Affiliations:** 1 Institute of Hematology, Medical College, Jinan University, Guangzhou, 510632, China; 2 Key Laboratory for Regenerative Medicine of Ministry of Education, Jinan University, Guangzhou, 510632, China; 3 Department of Hematology, Guangdong Province People's Hospital, Guangzhou 510080, China; 4 Department of Hematology and Oncology, Ernst-Moritz-Arndt University Greifswald, Greifswald 17487, Germany; 5 Institute of Human Genetics, Polish Academy of Sciences, Poznan, Poland

## Abstract

**Background:**

T-cell immunodeficiency is a common feature in cancer patients, which may relate to initiation and development of tumor. Based on our previous finding, to further characterize the immune status, T cell proliferative history was analyzed in CD4+ and CD8+ T cells from chronic myeloid leukemia (CML) patients.

**Methods:**

Quantitative analysis of δRec-ψJα signal joint T cell receptor excision circles (sjTRECs) was performed in PBMCs, CD3+, CD4+ and CD8+T cells by real-time PCR, and the analysis of 23 *TRBV-D1 *sjTRECs was performed by semi-nested PCR. Forty eight CML cases in chronic phase (CML-CP) were selected for this study and 17 healthy individuals served as controls.

**Results:**

The levels of δRec-ψJα sjTRECs in PBMCs, CD3+, CD4+, and CD8+ T cells were significantly decreased in CML patients, compared with control groups. Moreover, the numbers of detectable *TRBV *subfamily sjTRECs, as well as the frequency of particular *TRBV-BD*1 sjTRECs in patients with CML were significantly lower than those from healthy individuals.

**Conclusions:**

We observed decreased levels of recent thymic emigrants in CD4+ and CD8+ T cells that may underlay the persistent immunodeficiency in CML patients.

## Background

Chronic myeloid leukemia (CML), with the incidence of 1.5/100,000 population, represents 15% of newly diagnosed leukemia cases in adults in China. The prognosis in CML improved markedly after introduction of abl tyrosine kinase inhibitors (Immatinib mesylate and its derivatives), still a lot of CML patients die due to abl mutation related drug resistance and the blast crisis [1]. Therefore further studies are needed in order to better understand the disease and to improve the patient outcome. T cell immunodeficiency was suggested to play an important role in tumor progression, facilitating the expansion of the malignant clone [2,3], although the interaction between the tumor and the immune system is still not completely understood.

Most circulating mature T-cells use the α/β heterodimeric T cell receptor (TCR) for specific recognition of antigenic peptides in context of major histocompatibility complex (MHC) molecules. T cell differentiation in the thymus is characterized by a hierarchical order of rearrangement steps in the TCR genes, resulting in the joining of one of multiple variable (V), diversity (D), and joining (J) gene segments. This results in each differentiating T cell expressing unique TCR on the surface. The *TCR *beta locus (*TRB*) contains at least 64 functional V genes (*TRBV*) subdivided into 24 families [4]. In addition to the formation of the V(D)J coding joint, each of the *TCR *rearrangement steps generates circular episomal DNA fragments - signal joint T cell recombination excision circles (sjTRECs). During the process of TCR alpha-delta locus (*TRAD*) rearrangement, the TCR delta gene (*TRD*), which is located within the TCR alpha gene (*TRA*), has to be deleted before the *TRA *recombination starts. Rearrangement between two *TRD *deleting elements, δRec and ψJα, produces a δRec-ψJα signal joint TRECs [5-9]. sjTRECs are assumed to have a high over-time stability, but they can not multiply and consequently are diluted during T cell proliferation. A maximum of two sjTRECs can be present within one αβ T cell if the corresponding rearrangement event occurs in both alleles and if the cell did not divide upon the rearrangement. sjTRECs are exported from thymus to the periphery within recent thymic emigrants (RTEs), therefore, the frequency of sjTRECs is considered to be the most accurate marker of T-cell neogenesis. Quantitative detection of sjTRECs can be applied for direct measurement of thymic output and proliferative history of T cells [6]. Over the last decade the technique was used to evaluate T-cell immune reconstitution in different immunodeficiency diseases [6,10-13]. To assess the proliferative history in different *TRBV *subfamilies of T cells, quantitative analysis of *TRBV-BD *sjTRECs has been developed [12,14,15].

The first sjTREC analysis in hematopoietic malignancy was reported by Petridou et al [16], who compared the sjTREC values in childhood B-ALL and T-ALL. Significant reduction of sjTREC values was observed in T-ALL, whereas children with B-ALL had slightly but insignificantly lower sjTRECs values compared with healthy controls. In another study, consistent with the reduction of naïve T cells, thymopoiesis (measured by sjTRECs levels) was significantly lower in 73 children with ALL than in normal controls [17]. However, little data exist regarding the proliferative history of T cells in myeloid leukemia patients. Recently, we published the first analysis of the sjTRECs-content in patients with acute myeloid leukemia (AML) [18]. Our previous study showed decreased δRec-ψJα sjTRECs level and skewed *TRBV *repertoire in peripheral blood mononuclear cells (PBMCs) from 20 CML cases [19]. Since the high number of CML cells in the blood might have influenced the results, in the present study, in order to more precisely characterize the immune status in chronic myeloid leukemia (CML), we analyzed both δRec-ψJα sjTRECs and *TRBV-BD *sjTRECs in sorted CD4+ and CD8+ T cells from CML patients.

## Materials and methods

### Samples

Forty eight newly diagnosed chronic phase CML patients, 33 males and 15 females (13-81 years old; median age: 30 years) were included in this study. BCR-ABL fusion gene was detected in all samples by RT-PCR. Seventeen healthy individuals: 6 males and 11 females (25-51 years old, median age: 28 years) served as controls. The samples were collected at Dept. of Hematology, Guangdong Province People's Hospital, all the procedures were conducted according to the guidelines of the Medical Ethics committees of the health bureau of Guangdong Province of China. sjTRECs were measured in PBMCs from all 48 cases, and CD4+ and CD8+ T cells from 19 cases. *TRBV *sjTRECs were determined in PBMCs, CD4+ and CD8+ T cells from 10 patients. The clinical data of the patients are listed in Table 1.

**Table 1:** Clinical data of CML patients

No.	sex	age	WBC (×10^9^/L)	Blast+promyelocyte cells (%)	Platelets (×10^9^/L)	CD3+%	CD4+/CD8+ cells sorted
C1	F	49	213.59	9	147	28.91	Yes
C2	M	16	351.16	0	345	4.71	Yes
C3	M	58	59.93	6	144	11.01	Yes
C4	M	20	124	0	605	18.2	Yes
C5	M	25	256.82	6	109	13.8	Yes
C6	M	15	333.95	8.5	208	10.8	Yes
C7	F	31	294.91	3	252	10.46	Yes
C8	M	30	118.55	5	440	9.6	Yes
C9	M	24	244.05	9	750	12.04	Yes
C10	M	61	279	10	993	11.8	Yes
C11	F	30	99.8	6.5	378	2.1	Yes
C12	M	38	103.66	1	181	10.4	Yes
C13	F	20	450.45	1	396	12.1	Yes
C14	M	42	81.6	6	85	14.18	Yes
C15	M	73	196	8	1531	9.1	Yes
C16	M	31	129	3.5	285	28.4	Yes
C17	M	22	76.6	5.5	171	19.64	Yes
C18	M	20	112.7	5	596	42.5	Yes
C19	F	19	7	4	125	28.0	Yes
C20	M	61	44.9	12	77	13.6	No
C21	F	13	314.78	3	640	32	No
C22	M	59	18.54	2	695	56.89	No
C23	M	50	31.5	0	163	36.51	No
C24	M	35	5.1	5	283	38.6	No
C25	F	66	62.87	2	657	7.8	No
C26	F	30	160	16	842	12.4	No
C27	F	26	114.17	2	222	19.1	No
C28	M	26	5.3	0	118	44.7	No
C29	M	15	185.9	3	291	10.5	No
C30	M	27	101.5	3	326	42.5	No
C31	F	21	29.7	2	296	26.67	No
C32	M	22	111.92	0	115	9.17	No
C33	F	75	267	7	258	11.2	No
C34	M	29	0.08	2	34	18.75	No
C35	M	26	61.67	9	661	31.5	No
C36	M	43	170	0	671	40.66	No
C37	M	36	43.87	6	69	32.07	No
C38	M	38	58.55	0	3363	18.95	No
C39	M	29	132.4	10.5	1221	26.9	No
C40	F	55	130.21	4	204	14.2	No
C41	M	44	485.1	1	514	27.2	No
C42	F	16	1.39	0	46	38.2	No
C43	F	35	102.85	1	335	41.7	No
C44	F	25	33.44	6	470	35.0	No
C45	M	81	30.3	0	747	9.02	No
C46	M	38	154	1	485	13.75	No
C47	M	30	2.32	0	46	41.9	No
C48	M	25	7.63	11	139	49.3	No

### Mononuclear cells isolation

Peripheral blood mononuclear cells (PBMCs) were isolated from CML patients and healthy individuals by Ficoll-Hypaque gradient centrifugation.

### CD3+ cells determination

CD3+ T cells percentage from PBMCs was determined by indirect immune fluorescent analysis. The PLP-fixed cytospin preparations were incubated with 200 μg/ml of murine anti-CD3 mAb (Boster Biological Technology Ltd, Wuhan, China), washed and incubated with 1:50 dilution of fluorescein labeled goat anti-mouse Ig (Boster Biological Technology Ltd, Wuhan, China). The slides were counterstained with Mayer's hematoxylin for 30 min. All slides were blindly evaluated using the fluorescent microscope (Nikon WFX-II, Nikon Ltd, Japan); 200 cells were counted.

### T cells sorting

The CD4+ and CD8+ T cells from 19 CML cases and 17 healthy individuals were sorted using CD4 and CD8 monoclonal antibody and MACS^® ^Magnetic Cell sorting technique (Miltenyi Biotec, Bergisch Gladbach, Germany). After CD4+ and CD8+ T cells sorting, the purity was determined by indirect immune fluorescent analysis. The positive cells were around 95% to 97%.

### DNA extraction

Total DNA from distinct cell populations was extracted using QIAamp^® ^DNA Blood Mini Kit (QIAGEN, Germany), the quality of RNA was analyzed in 0.8% agarose gel stained with ethidium bromide and the concentration was determined by spectrophotometric analysis at 260 and 280 nm (Lambda 45 UV/VIS Spectrometer, Perkin Elmer USA).

### Real-time quantitative PCR (RQ-PCR)

Quantitative detection of δRec-ψJα sjTRECs in DNA from PBMCs and sorted CD4+ or CD8+ T cells was preformed by real-time PCR using the ABI PRISM 7700 Sequence Detector TaqMan (PE Biosystems, Foster City, CA). PCR was performed as described by previous studies [15,20]. To precisely determine the percentage of cells carrying sjTREC we constructed a duplex vector including a fragment of the δRec-ψJα (sjTREC) and a fragment of the RAG2 gene used as a reference. The RAG2 was cloned first in the T-A acceptor site and subsequently the sjTREC was cloned in to the EcoRV restriction site of the TOPO TA Vector (Invitrogen, Groning, The Netherlands). Based on the DNA concentration, measured by spectrophotometry and confirmed by a quantitative gel eletrophoresis, standard dilutions of the vector from 10^7 ^to 10^1 ^copies were prepared [15,20]. In brief, PCR of 25 μl total volume was performed with approximately 100 ng of genomic DNA, 25 pmol of each primers (TREC-1 and TREC-2 for sjTRECs, RAG2-for and RAG2-back for RGA2 amplification), 10 nmol each dNTP, 1.5 U AmpliTaq Gold (Applied Biosystems, Branchburg, New Jersey, USA), 5 pmol of 6FAM-TAMRA probe and PCR Buffer including 4.5 mM MgCl_2_. After the initial denaturation at 95°C for 5 min, 45 cycles consisting of 95°C for 30 sec and 67°C for 1 min were performed. If no TRECs were detected in a sample, PCR was repeated with more DNA.

### TRBV-BD1 sjTRECs detection by semi-nest PCR

Twenty three *TRBV-BD*1 sjTRECs were amplified by semi-nest PCR from different amounts of genomic DNA (1.3 μg, 325 ng or 65 ng, corresponding to 2 × 10^5^, 5 × 10^4 ^or 1 × 10^4^cells respectively) isolated from PBMCs, CD4+ and CD8+ T cells. Two nested 5' *TRBD*1 primers, located upstream of the segment, and twenty three 3' *TRBV *primers (*BV*1-19 and *BV*21-24) were used [15,20]. Since the *TRBV*20-*BD*1 rearrangement occurs by inversion, it does not generate a sjTREC. In the first round PCR, 2 μl of genomic DNA were amplified in a 10 μl reaction mixture containing: 0.375 μM external sense and antisense primers, 0.1 mM dNTP, 1.5 mM MgCl_2_, 1× PCR buffer and 1 U Taq polymerase (GoTaq^® ^Flexi DNA polymerase, Promega, Madison, WI, USA) using the DNA thermal cycler. After 3 min denaturation at 94°C, 30 PCR cycles were performed, each cycle consisting of 94°C for 1.5 min, 65°C for 1 min and 72°C for 1 min, and a final 6 min elongation at 72°C. Then, the products were stored at 4°C. In the second round PCR, 25 cycles of amplification were carried out with 2 μl of the first PCR products, the same BV primer and the internal sense BD1 primer.

### Statistical analysis

Univariate analyses were done using the Mann-Whitney test to compare the numbers of δRec-ψJα sjTRECs and detectable *TRBV-BD*1 sjTRECs in CML and healthy control groups. The chi square test was used to compare the frequency of *TRBV-BD*1 sjTRECs in PBMCs in CML and healthy control groups. Pearson correlation and linear regression analysis were used to estimate the correlation between age and sjTRECs numbers.

## Results

### Decreased level of δRec-ψJα sjTRECs in PBMCs, CD4+ and CD8+ cells from CML patients

**Figure 1:**
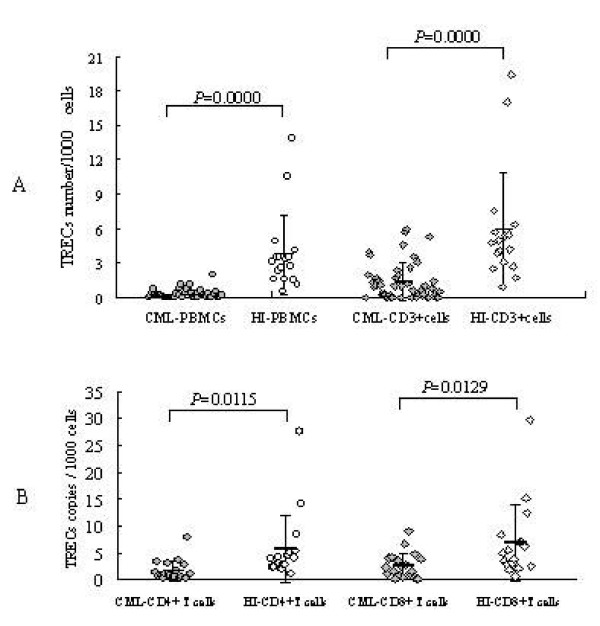
**Comparison of the sjTRECs levels in patients with CML and healthy individuals (HI)**. A: The sjTRECs levels in PBMCs; B: The sjTRECs levels in CD4+ and CD8+ T cells respectively.

The absolute numbers of sjTRECs and RAG2 were measured in two independent assays and sjTREC content per 1000 PBMCs was calculated using a formula n = 2 × 1000 × [sjTREC(1)+sjTREC(2)]/[RAG2(1)+RAG2(2)] [15]. The absolute numbers of sjTRECs in T cells were determined by the percentage of CD3-positive cells (n = sjTRECs/1000 PBMCs÷CD3^+^%). The CD3+ percentage in PBMCs from healthy individuals was 62.32 ± 4.72%, and 22.89 ± 13.76% in PBMCs from CML patients. The sjTRECs levels in PBMCs, CD3+, CD4+ and CD8+ T cells from patients with CML are shown in Figure 1. In comparison with the sjTRECs in healthy individuals (3.76 ± 3.42 copies/1000 PBMCs, 5.87 ± 4.96 copies/1000 CD3+ cells, 5.62 ± 6.45 copies/1000 CD4+ T cells, 6.79 ± 7.1 copies/1000 CD8+T cells), a dramatic reduction of sjTRECs values was found in patients with CML (0.23 ± 0.38 copies/1000 PBMCs, 1.34 ± 1.63 copies/1000 CD3+ cells, 1.49 ± 1.88/1000 CD4+ T cells, 2.52 ± 2.43 copies/1000 CD8+ T cells) (p < 0.0001, p < 0.0001, p = 0.0115 and p = 0.0129, respectively).

The numbers of sjTRECs in PBMCs and sorted T cells from CML were higher in females than in males. The values were: the PBMCs group: 0.19 ± 0.25 copies/1000cells in male (n = 33) versus 0.43 ± 0.56 copies/1000cells in female (n = 15) (p = 0.0467), in the CD3+T cells group: 1.05 ± 1.21 copies/1000cells in male (n = 33) versus 1.97 ± 2.25 copies/1000cells in female (n = 15) (p = 0.0712), in the CD4+T cells group: 1.4 ± 2.08 copies/1000cells in male (n = 14) versus 1.74 ± 1.31 copies/1000cells in female (n = 5) (p = 0.739), and in the CD8+T cells group: 1.66 ± 1.63 copies/1000cells in male (n = 14) versus 4.95 ± 2.82 copies/1000cells in female (n = 5) (p = 0.0053). Similar results were found in healthy individual group (data not shown). Although the differences between genders were quite obvious, they were not statistically significant, except for PBMCs and CD8+ cells in CML patients.

### Lower frequencies of 23 *TRBV-BD1 *sjTRECs in PBMCs, CD4+ and CD8+ cells from CML patients

The *TRBV-BD*1 sjTRECs from *TRBV*1-19 and *TRBV*21-24 were analyzed by semi-nested PCR, using different amounts of DNA (corresponding to 2 × 10^5^, 5 × 10^4^or 1 × 10^4 ^cells respectively). Samples were amplified to estimate the frequency of TCR *TRBV-BD1 *sjTRECs and the sequences of the junction regions of each *TRBV-BD1 *sjTRECs were confirmed by PCR products direct sequencing (data not shown).

**Figure 2:**
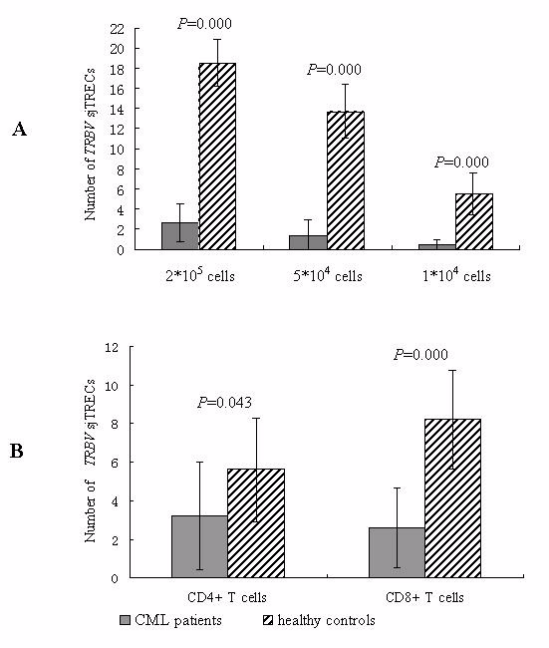
**The number of detectable subfamilies of *TRBV-BD1 *sjTRECs in from CML patients and healthy controls**. A: The subfamily numbers of *TRBV-BD1 *sjTRECs in PBMCs; B: The subfamily numbers of *TRBV-BD1 *sjTRECs in CD4+ and CD8+ T cells (1 × 10^4 ^cells) respectively.

**Figure 3:**
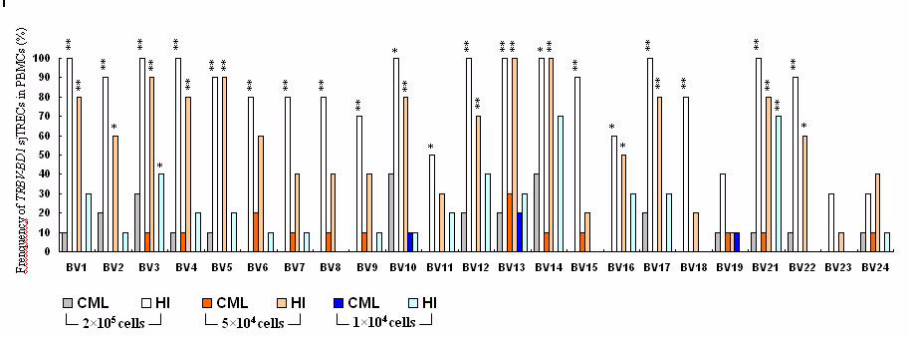
**Comparison the frequencies of 23 *TRBV-BD1 *sjTRECs in PBMCs between CML patients and healthy controls (HI) at different amounts of DNA level (n = 10)**. Note: *: compare to normal control p < 0.05, **: compare to normal control p < 0.01.

**Figure 4:**
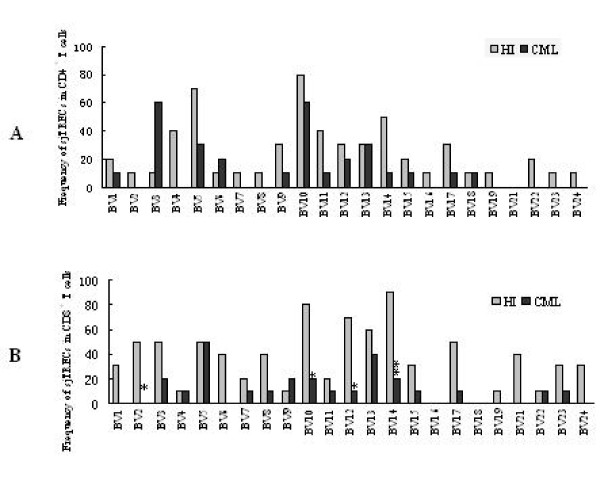
**Comparison the frequencies of 23 *TRBV-BD1 *sjTRECs in CD4+ (A) and CD8+T cells (B) between CML patients and healthy controls (HI) (n = 10)**. Note: *: compare to normal control p < 0.05, **: compare to normal control p < 0.01.

The number of detectable *TRBV *subfamily sjTRECs differed significantly between CML and healthy control in 2 × 10^5^, 5 × 10^4 ^and 1 × 10^4 ^PBMCs or in 1 × 10^4 ^of CD4+ and CD8+ T cells (Figure 2). Comparison of the frequencies of 23 *TRBV-BD1 *sjTRECs in PBMCs between CML patients and normal controls at different amounts of DNA level showed that the frequencies of the most *TRBV *subfamily sjTRECs were significantly lower than those from healthy individuals, especially at the higher cellular concentration (2 × 10^5 ^PBMCs) (Figure 3). But the significant difference was found only in few subfamilies (*BV*2, *BV*10, *BV*12 and *BV*14 in CD8+T cells) when comparing the frequency of *TRBV *subfamily sjTRECs in CD4+ and CD8+ T cells at 1 × 10^4 ^concentration between both group (Figure 4).

## Discussion

In patients with CML, cellular immune deficiency is a common feature which may be due to decreased output of recent thymic emigrants, the abnormal expression of T cell receptor repertoire and, may in part, due to altered expression of TCR-CD3 complex. Our previous study showed decreased δRec-ψJα sjTRECs level and skewed *TRBV *repertoire in peripheral blood mononuclear cells (PBMCs) from CML patients [19,21]. And TCR ζ chain expression was decreased in T cells from patients with CML [22,23].

In order to further evaluate the T-cell immune function, the T cell proliferative history in CML patients was analyzed. The sjTRECs-content in PBMCs and CD3+ T cells from 48 CML cases was determined. The results confirmed our previous smaller study [19]. We showed a dramatic reduction of sjTRECs values in CML patients. In some cases no sjTRECs could be detected in 40 000 T cells. This suggests poor thymic output in CML patients, which may be even more pronounced than in ALL patients [16]. To date there are only a few papers describing TRECs level in hematopoietic malignancies [16,17]. The exact value of sjTRECs level in PBMCs from CML patients are influenced by contaminating normal non-T cells and leukemia blast cells; therefore the sjTRECs numbers were normalized with the percentage of CD3+cells in the analyzed samples. Furthermore, we analyzed sjTRECs in sorted CD4+ and CD8+ T cells. This is the most sensitive and accurate method for quantitation of naïve T-cells. It allows also the comparison of sjTRECs levels in CD4+ and CD8+ subsets. The levels of sjTRECs-expressing CD4+ and CD8+ T cells were significantly decreased in CML patients, as compared with age and sex matched healthy individuals. The decrease of sjTRECs levels was similar in both T cell subsets. These findings suggest that an impaired thymic output function and, as a consequence, an altered ability to maintain T cell homeostasis, which may play an important role in the immunodeficiency in CML patients. However, whether this is due to the clonal expansion of T-cells to antigens, for example leukemia associated antigens, or reflects the impairment of immune function associated with the malignancy, remains an open question [7,24-27].

Pido-Lopez et al showed that the decline in number of recent thymic emigrants in the blood with increasing age is gender-linked [28]. Peripheral blood from female contained significantly higher levels of sjTRECs per CD3+ T cell than blood from males. Also in children, the number of sjTRECs was higher in healthy girls than in healthy boys, and a similar pattern was evident in T-cell malignancies [16]. In the present study, we observed slightly, but in-significantly higher sjTRECs levels in healthy females, however, the number of sjTRECs was statistically higher in PBMCs and CD8+ T cells from female CML patients.

The majority of studies published previously focused only on the total thymic output, as measured by quantitative analysis of δRec-ψJα sjTRECs [6]. This approach doesn't allow the evaluation of the complexity of thymic output in different *TRBV *subfamily naïve T cells, which is an important factor in immune competence. In this study, we analyzed the total 23 subfamilies of *TRBV-DB1 *sjTRECs in PBMCs, CD4+ and CD8+ T-cells from CML patients by a semi-nested PCR. The results indicate that the percentage of cases positive for *TRBV-DB1 *sjTRECs varies in different *BV *subfamilies in healthy controls; the highest for *TRBV1, 3, 4, 10, 12-14, 17 *and *V21*, which could be detected in all 10 samples (at 2 × 10^5 ^PBMCs). The most important observation in this study was the significantly lower frequency of 23 *TRBV-BD*1 sjTRECs in PBMCs, as well as in CD4+ and CD8+ T cells from CML patients as compared with healthy individuals, indicating poor thymic output in CML patients. The results further support and explain the significant reduction of recent thymic emigrant numbers in peripheral blood of CML patients, as measured by quantitative detection of δRec-ψJα sjTRECs.

In conclusion, this is, to our knowledge, the first characterization of thymic output function in CD4+ and CD8+ T cells from CML patients based on analyses of both δRec-ψJα sjTRECs and *TRBV-DB1 *subfamily specific sjTRECs. We showed a prominent decrease of sjTRECs levels in CML, indicating the reduction of recent thymic emigrants affects the majority of *TRBV *subfamilies.

## Competing interests

The authors declare that they have no competing interests.

## Authors' contributions

YQL, CAS and GKP were responsible for study design and data management. SXG and SHC performed the real-time PCR, QSY and LJY performed the semi-nested PCR, XLW and BL performed the statistical analysis, XD collected samples. All authors read and approved the final manuscript.
